# Supported Ultra-Thin Alumina Membranes with Graphene as Efficient Interference Enhanced Raman Scattering Platforms for Sensing

**DOI:** 10.3390/nano10050830

**Published:** 2020-04-27

**Authors:** Montserrat Aguilar-Pujol, Rafael Ramírez-Jiménez, Elisabet Xifre-Perez, Sandra Cortijo-Campos, Javier Bartolomé, Lluis F. Marsal, Alicia de Andrés

**Affiliations:** 1 Instituto de Ciencia de Materiales de Madrid, Consejo Superior de Investigaciones Científicas Cantoblanco, 28049 Madrid, Spain; xochitl.aguilarpujol@gmail.com (M.A.-P.); ramirez@fis.uc3m.es (R.R.-J.); s.cortijo@csic.es (S.C.-C.); jbvilchez@gmail.com (J.B.); 2 Departamento de Física, Escuela Politécnica Superior, Universidad Carlos III de Madrid, Avenida Universidad 30, Leganés, 28911 Madrid, Spain; 3 Department of Electronic Engineering, Universitat Rovira i Virgili, Avda. Països Catalans 26, 43007 Tarragona, Spain; elisabet.xifre@urv.cat (E.X.-P.); lluis.marsal@urv.cat (L.F.M.); 4 Departamento de Física de Materiales, Universidad Complutense de Madrid, Plaza Ciencias 1, 28040 Madrid, Spain

**Keywords:** interference, enhanced Raman scattering, alumina membrane, graphene, nanoparticles, optical simulations, AFM, SEM

## Abstract

The detection of Raman signals from diluted molecules or biomaterials in complex media is still a challenge. Besides the widely studied Raman enhancement by nanoparticle plasmons, interference mechanisms provide an interesting option. A novel approach for amplification platforms based on supported thin alumina membranes was designed and fabricated to optimize the interference processes. The dielectric layer is the extremely thin alumina membrane itself and, its metallic aluminum support, the reflecting medium. A CVD (chemical vapor deposition) single-layer graphene is transferred on the membrane to serve as substrate to deposit the analyte. Experimental results and simulations of the interference processes were employed to determine the relevant parameters of the structure to optimize the Raman enhancement factor (E.F.). Highly homogeneous E.F. over the platform surface are obtained, typically 370 ± (5%), for membranes with ~100 nm pore depth, ~18 nm pore diameter and the complete elimination of the Al_2_O_3_ bottom barrier layer. The combined surface enhanced Raman scattering (SERS) and interference amplification is also demonstrated by depositing ultra-small silver nanoparticles. This new approach to amplify the Raman signal of analytes is easily obtained, low-cost and robust with useful enhancement factors (~400) and allows only interference or combined enhancement mechanisms, depending on the analyte requirements.

## 1. Introduction

The search of novel platforms for the amplification of Raman signal is still an objective since Raman spectroscopy is one of the most powerful techniques to identify analytes through the characteristic vibration modes of molecules and crystals. The detection and imaging of extremely diluted and/or complex materials still require further research and development to get cheap, reliable, reproducible and stable over time systems that can be easily reused several times. The enhancement achieved using localized plasmons, the so-called surface enhanced Raman scattering (SERS) [1,2,3,4] is definitely the most efficient process allowing to reach single molecule detection through complex structures and strategies [3,5,6]. Nevertheless, reproducibility, stability, complexity and reusability are still issues [7]. Another crucial challenge is obtaining homogeneous enhancement across the platforms to allow reliable quantitative detection [8]. Recently, the fabrication of hybrid platforms that combine nano- or micro-structured semiconductors (nanopillars, nanorods, etc.) with metallic nanoparticles (NPs) has been proposed to further enhance amplification [9,10]; however, homogeneity is achieved by employing expensive lithographic methods. Another method proposed to increase the specific area is by the formation of nanoporous metals [11].The combination of metallic NPs or nanostructures with graphene is an interesting approach since graphene may also provide an extra enhancement of chemical nature, the chemical mechanism (CM) [9,12]. A related but different approach consists in the deposition of nanostructured metallic arrays coupled with metallic films separated by a very thin dielectric spacer layer [13] as the case of gold nanopyramid arrays coupled to a gold film separated by a silica layer, which led to strong light absorption confined in the space layer with E.F. up to 233 [14]. The high enhancement of plasmon intensity at the gap is of interest because of its applications in metamaterials, energy transfer, sensors and solar energy harvesting [15].

In this context, the amplification of the electromagnetic signal based on interference processes has been scarcely explored. The interference of light occurring at the interfaces of multilayered heterostructures that combine materials with very different refractive indices (*n*) can be tuned depending the application. The interference enhanced Raman scattering (IERS) was applied in the 1980s to detect the phonons of ultrathin films [16,17] obtaining enhancement factors (E.F.) of around 20. It is now commonly used to increase the Raman signal of graphene and other 2D materials typically by using a SiO_2_ dielectric layer on silicon single crystals with gains up to around 40 [18,19,20,21]. E.F. of up to 70 was reported for graphene bubbles on copper [22]. Some examples can be found on the combination of interference and SERS mechanisms [23,24] with low amplification factors, and, recently the design of optimized amplification platforms using aluminum as reflecting surface and Al_2_O_3_ as dielectric layer demonstrated efficient IERS and combined IERS and SERS amplifications [25]. 

Here we propose a new concept for an interference amplification platform which is robust and versatile. It is based on supported porous alumina membranes where the dielectric layer is the alumina membrane and the air of its pores and the reflecting layer is the metallic aluminum foil at the base of the membrane. Graphene is transferred on top of the porous alumina membrane and serves as the support where the analyte is deposited. Graphene is an excellent bio-compatible platform [26,27,28] with interesting characteristics since it can by-pass metal-biomaterial interactions occurring in SERS and quenches molecular fluorescence, highly inopportune for Raman spectroscopy. We have studied the different parameters that control the amplification factor such as the depth and density of the pores or the presence of an alumina barrier at the bottom of them. Interference is extremely sensitive to the thickness of the dielectric layer, which in this case is the depth of the pores but still we obtained highly efficient IERS with factors up to 400 and homogeneous amplification over the sample surface. 

## 2. Materials and Methods

Porous alumina was obtained by a two-step electrochemical anodization process of aluminum to obtain a porous alumina layers with highly ordered pore distribution [29]. Before any anodization, pure aluminum substrates (99.999% purity) were cleaned with acetone, water and ethanol and electropolished in a mixture of ethanol (EtOH) and perchloric acid (HClO_4_) 4:1 (*v*/*v*) at 20 V for 5 min in order to eliminate the surface roughness of the commercial aluminum substrates [30]. Subsequently, the first electrochemical anodization step took place in an aqueous solution of sulphuric acid (H_2_SO_4_) 0.3 M at 10 V and 3 °C. After 20 h, an alumina layer with disordered pores was obtained. It was dissolved by wet chemical etching in a mixture of 0.4 M phosphoric acid (H_3_PO_4_) and 0.2 M chromic acid (H_2_CrO_7_) at 70 °C for 3 h [31]. The second anodization step was performed under the same conditions as the previous one. The anodization time of this step determined the pore depth of the final ordered pore alumina layer [32]. The above mentioned combination of sulphuric acid, anodization voltage and electrolyte temperature has been specially selected for obtaining ultrathin alumina layers. With this selection we manage to extraordinarily slow down the speed of the anodization process and fabricate extremely thin layers with controlled thickness. Finally, the barrier layer formed at the bottom of the pores, inherent to the electrochemically obtained alumina, needed to be eliminated to increase the E.F. of the designed platforms. A simple and rapid method for its elimination was developed for these extremely small pores that consisted of the steady decrease of the anodization current followed by a pore-widening treatment performed by wet chemical etching in H_3_PO_4_ 5 wt % at 35 °C for 4.5 min.

PMMA (polymethilmetacrylate)/Graphene on copper foil from *Graphenea* company (San Sebastián, Spain) was transferred onto the porous alumina. Cu was first eliminated in a 2.1 M FeCl_3_ and 1.3 M HCl solution for 15 min. The PMMA/Graphene stack was rinsed in a deionized water bath twice, immerged in a 10% HCl solution and then again into deionized water three times with the last bath for 20 h to complete the removal of Cu. Subsequently, the Gr/PMMA film was fished onto the porous alumina samples and baked on a hot plate at 90 °C. PMMA was then removed by immersing in warm acetone at 50 °C followed by further vacuum thermal treatment at 250 °C. 

Silver nanoparticles were deposited at room temperature with the gas aggregation technique [4,33] using a magnetron sputtering source (Nanogen50, from Mantis Ltd., Manchester, United Kingdom) and an Ag target 99.95% purity. The ejection of atoms and nucleation of clusters is assisted by a mixture of Ar/He gas, carried along the aggregation chamber through an orifice and reaching the substrate. The base pressure was 5 × 10^−9^ mbar and the work pressures were 2.5 × 10^−1^ mbar inside the aggregation chamber and 2 × 10^−3^ mbar in the deposition chamber, with an Ar/He ratio of 1:2.4. These parameters give rise to spherical single crystalline nanoparticles with average diameter ~4 nm [34]. The Ag NPs were simultaneously deposited on the membrane/graphene and on the fused silica/graphene reference sample. Rhodamine 6G (R6G) films were then deposited by spin coating from a methanol solution (10^−3^ M) on the two previous samples (membrane/graphene/AgNPs and fused silica/graphene/AgNPs). 

Micro-Raman experiments were performed at room temperature with the 488 nm line of an Ar^+^ laser, incident power in the 0.1–8 mW range, an Olympus microscope (×100 and ×20 objectives) and a “super-notch-plus” filter from Kaiser. The scattered light was analyzed with a Horiba monochromator coupled to a Peltier cooled Synapse CCD. The estimated Raman spatial resolution is around 0.7 µm at 488 nm for the high NA (0.95) ×100 objective. The Raman signal of graphene transferred on fused silica is used as the reference to calculate the enhancement factors. Raman signal of single layer graphene is very regular over any standard substrate so the average of three measurements was used.

The morphology and roughness of the samples were examined using atomic force microscopy (AFM) (equipment and software from Nanotec^TM^, Madrid, Spain) [35]. Topographic characterization was carried out in the tapping mode, using commercial Si tips (Nanosensors PPP-NCH-w) with a cantilever resonance frequency *f*_0_ ≈ 270 kHz and *k* ~ 30 Nm^−1^. Several regions were probed to confirm homogeneity of the surfaces to the micrometer scale. An estimation of the roughness is obtained from the full width at half-maximum (FWHM) of the height distribution of the analyzed topographic images. 

The simulations of the Raman signal amplification in the membranes were performed calculating the propagation of light through multilayered media using the matrix transfer method. In this method, the amplitude of the electromagnetic waves at two different depths inside the structure are related by a complex matrix—the transfer matrix is constructed taking into account the geometry and the refractive indexes of each layer, the effect on the electromagnetic (EM) field amplitude when traveling through different layers is calculated by matrix multiplication. The method provides a solution to Maxwell equations that considers the interference of the infinite number of multiple reflections occurring for light propagating through multilayered media. In the particular case of Raman scattering, multiple interference impacts in two ways, firstly the amplitude of the light arriving at a particular location in the structure, where the scattering process takes place should be calculated, secondly the scattered light should travel to the detector outside the structure, the calculation of the intensity involves again the transfer matrix method. Both aspects are taken into account in the calculations carried out previously for different metals and dielectrics [25]. 

## 3. Results and Discussion

The minimum requirement to obtain light multireflections able to produce interference is the presence of two parallel interfaces, one at the analyte to be detected and the other at a highly reflecting surface located at a certain distance, which can be tuned to obtain constructive or destructive interference.

**Figure 1:**
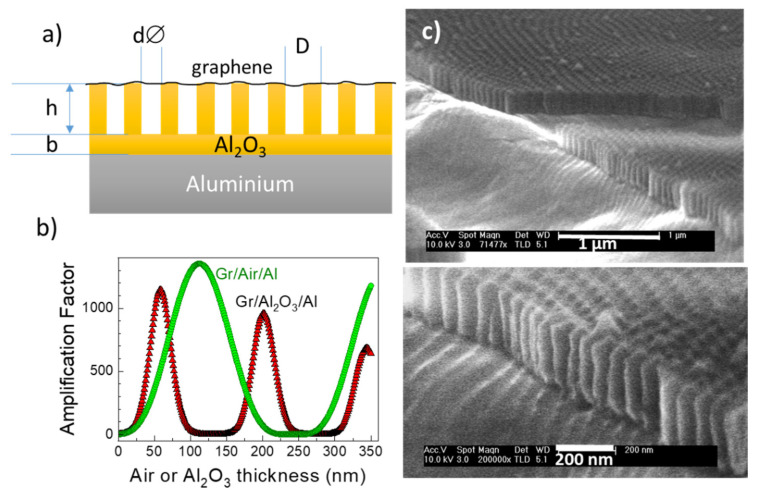
(**a**) Schema of the section of a supported membrane with a single layer graphene transferred on top of it, (**b**) calculated Raman amplification with Al_2_O_3_ (red triangles) or air (green circles) as the dielectric layer for 488 nm laser excitation, (**c**) scanning electron microscopy (SEM) images of an alumina supported membrane with *h* ≈ 200 nm.

The maximum interference for visible excitation lasers is obtained for aluminum as the reflecting layer, because of its high imaginary part *n_i_* (*n* = *n_r_* + *in_i_*), combined with a material with no absorption (*n_i_* ~ 0) and the smallest *n_r_* possible [25]. According to the simulations, air is the optimum dielectric medium, thus we have evaluated the amplification related to alumina membranes which are not detached from their aluminum base. Figure 1a sketches the section of a supported membrane with a single layer graphene transferred on top of it. The relevant parameters are indicated—the depth of the pore *h*, the diameter of the pore *d*, the distance between pores *D* and the thickness of the bottom Al_2_O_3_ barrier layer *b*. Figure 1b shows the calculated Raman amplification (for calculation details see Ref. [26]) for graphene phonons considering the simplest possible system where the dielectric layer is either Al_2_O_3_ (red triangles) or air (green circles). The ideal platform would thus include a dielectric layer formed by air with 120 nm thickness for 488 nm laser excitation. The supported membranes are an intermediate situation where air is present in a fraction of the dielectric layer, at the pores. The most Interference is strongly dependent on its thickness, in this case the depth of the pores, *h* in Figure 1a, so it is challenging to get enough control of this parameter in the fabrication process. Several sets of supported membranes were obtained with pore diameters, *d*, in the range 10–20 nm and different depths (*h*)—around 60, 100 and 200 nm (SEM images of the membrane with *h* ~ 200 nm is shown in Figure 1c). The presence of Al_2_O_3_ at the bottom of the pores is detrimental for the interference process so this barrier layer is reduced by a steady decrease of the anodization current followed by a pore-widening treatment, this later treatment attempts to increase the air fraction of the membrane to be closer to the ideal situation determined by the simulations.

**Figure 2:**
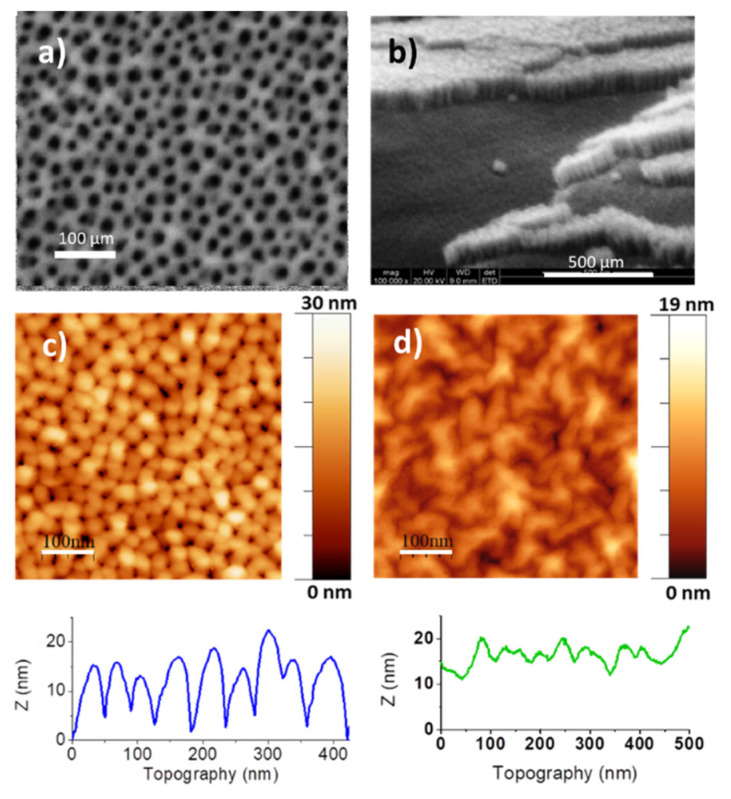
(**a**,**b**) SEM images of a membrane with pore diameter *d* ≈ 18 nm and *h* ≈ 100 nm, (**c**,**d**) atomic force microscopy (AFM) topographic images and height profiles of the pristine membrane and after graphene transfer, respectively.

Figure 2a,b show in-plane and tilted SEM images of a supported alumina membrane with pore depth around 100 nm (sample named *h* = 100 nm) and ~18 nm pore diameter. The transference of a single layer graphene on top of the membrane strongly modifies its AFM topographic image (Figure 2c,d). The analysis of the SEM and AFM images and the AFM profiles allow to estimate the pore to pore distance to be around 35 nm. An accurate determination of the pore diameter from the AFM images is challenging, since the tip size (20–30 nm) limits the estimation of pore lateral dimensions, however, obtaining pore to pore distance is quite precise. For the samples with transferred graphene, the profiles (and the height statistics) evidence how graphene mimics the membrane surface but strongly limits the oscillations to around ± 3 nm, much smaller than the pore depth (100 nm). The chosen small pore diameter, *d* < 20 nm, is therefore adequate to get small fluctuations of the overall dielectric thickness defined by the graphene top layer (the molecules to be sensed are deposited on top of the graphene).

**Figure 3:**
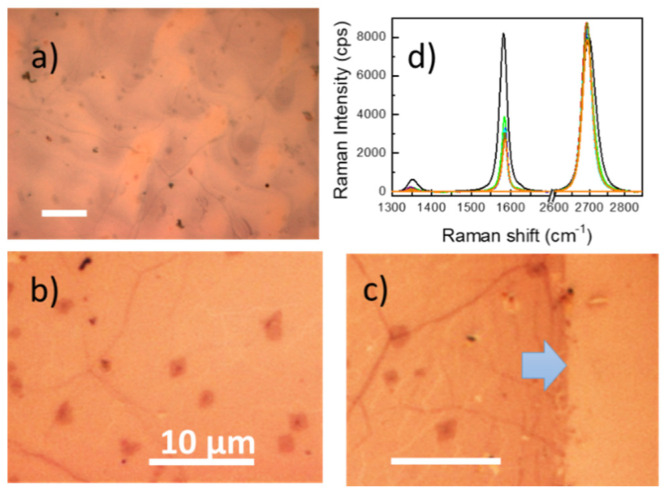
Optical images of an alumina supported membrane (*h* ≈ 100 nm) (**a**) with graphene/PMMA, (**b**) with graphene once PMMA is eliminated showing the wrinkles and bi/tri-layer graphene spots and (**c**) graphene edge (indicated by a blue arrow). (**d**) Raman spectra of graphene at different positions. Black curve corresponds to a graphene bilayer (darker spot in the images). The scale bar corresponds to 10 µm in all cases.

The graphene transfer process and final graphene quality are first checked by optical microscopy. Figure 3a corresponds to the alumina membrane after fishing the graphene/PMMA film. Once the process to eliminate PMMA is concluded, typical micron sized regions of bi/tri-layer graphene can be easily seen (darker micron-sized spots in the optical images) as well as graphene wrinkles (Figure 3b,c). The interference process can also increase the contrast of optical images, as it occurs here and it is the first indication that the system will provide Raman amplification. In Figure 3c the edge of the transferred single-layer graphene is easily seen showing its high quality up to the very edge. 

The quality of the transferred graphene is checked analyzing the Raman spectra. In Figure 3d the spectra of the *h* = 100 nm membrane at distant points are presented showing the characteristic D (≈1580 cm^−1^) and 2D (≈2700 cm^−1^) peaks with an intensity ratio *I*_2D_/*I*_G_ > 2 indicating the single layer character of the transferred graphene. A defect peak (D at ≈1350 cm^−1^) is detected with very small intensities. The black line in Figure 3d corresponds to a dark point in Figure 3b and clearly signals to a graphene bi-layer with the characteristic almost identical intensities of G and 2D peaks (*I*_G_ ≈ *I*_2D_), being *I*_G_ two times that of the single layer spectra and a higher intensity of the defect peak D. 

**Figure 4:**
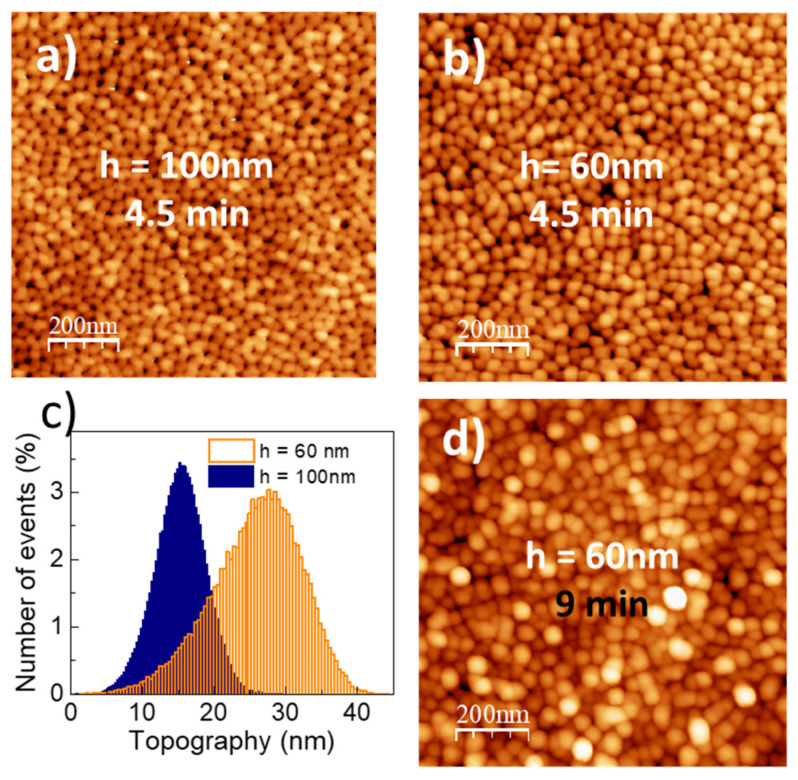
AFM images of (**a**) *h* = 100 nm (**b**) *h* = 60 nm *t* = 4.5 min and (**d**) *h* = 60 nm *t* = 9 min membranes. (**c**) Height distributions of the membranes in (**a**,**b**).

Membranes with *h* = 60 nm were fabricated to optimize the E.F., however, for such small pore depth the quality of the membranes is compromised in terms of the order of the pores as well as in the height uniformity. The AFM topographic images show an increased disorder of the pores for the *h* = 60 nm membrane compared to the *h* = 100 nm one (Figure 4a,b), also, the height distribution of the AFM image in the case of the 60 nm sample is increased significantly, especially in comparison with the total nominal hole depth (~25%) (Figure 4c). Attempts to increase the pore fraction by extending the pore-widening treatment time to 9 min, leads to further disorder (Figure 4d).

The efficiency of the interference process of the platforms is evaluated by measuring graphene Raman spectra compared to those of graphene transferred onto fused silica. The enhancement factor is defined as E.F. = *I*_2D_ (membrane)/*I*_2D_ (fused silica). The obtained amplification for the *h* = 60 nm *t* = 4.5 min is E.F. ≈ 265 and for *h* = 100 nm membrane, higher values are obtained with average value E.F. ≈ 370.

**Figure 5:**
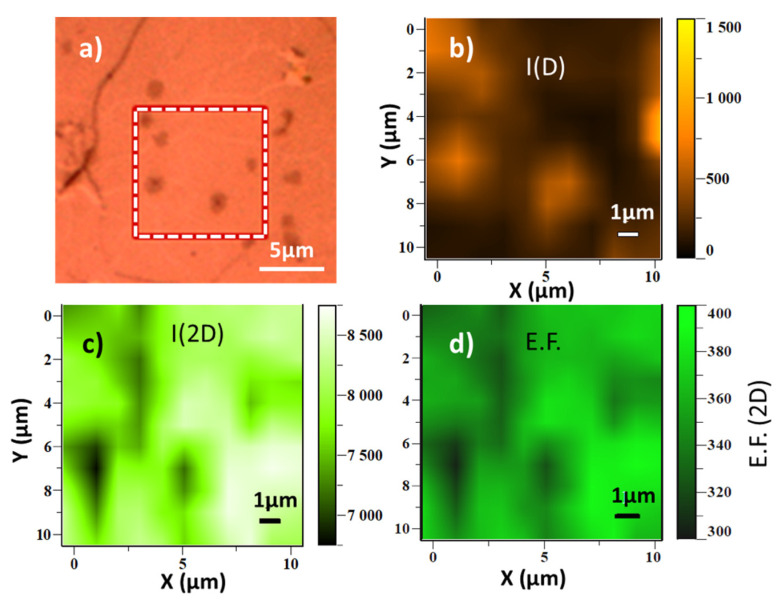
(**a**) Optical image of graphene on a 100 nm membrane, the 10 µm × 10 µm white dashed square is the tested area in the Raman images of (**b**) D peak intensity, (**c**) 2D peak intensity and (**d**) enhancement factor E.F. = *I*_2D_ (membrane)/*I*_2D_ (fused silica).

One important point is to evaluate the uniformity of the amplification over the sample at different scales. Therefore, besides recording a set of spectra at distant points, we obtained, for the *h* = 100 nm sample (optical image in Figure 5a), 121 spectra from 10 µm × 10 µm squares, 1 µm steps, that we can transform into Raman images of the 2D and D peak intensities (Figure 5b,c) and of the Raman enhancement factor E.F (Figure 5d). The 2D peak image (Figure 5b) shows a very uniform intensity with slightly lower intensity regions that correspond to the darker points of the optical image related to micron-sized areas of bi/tri-layer graphene (Figure 5a) as we commented before. Just the opposite occurs to the D peak whose intensity is increased at the bilayer graphene regions. Finally, the amplification, except at the small bilayer regions, is very uniform with an average value of 370 ± 5%. Such Raman amplification factors are remarkable and, interestingly, are obtained without the use of nanoparticles. 

**Figure 6:**
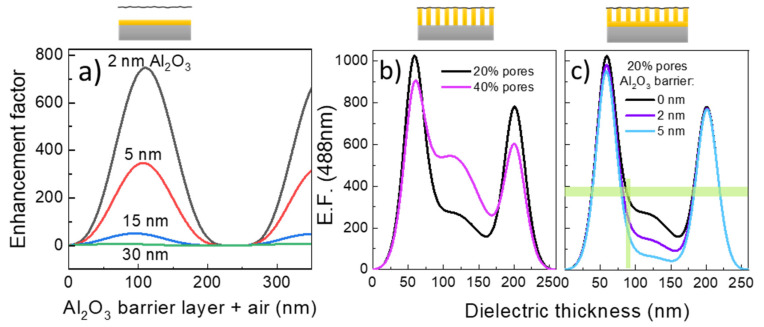
E.F. calculated at 488 nm laser excitation for (**a**) a dielectric formed by air and an Al_2_O_3_ barrier of the indicated thickness, (**b**) different pore fractions without barrier and (**c**) pore fraction of 20% and alumina barrier layer of 0, 2 and 5 nm. The horizontal green line corresponds to the experimental E.F. value.

Several characteristics of the membranes reduce the amplification for a perfect “air layer” obtained in the simulations. On one hand, the calculations indicate that an alumina barrier layer present at the bottom of the pores drastically depletes amplification (Figure 6a). In this figure the amplification is calculated as a function of the dielectric layer thickness, the dielectric being formed by air and different fixed alumina barrier layers (thickness: 2, 5, 15 and 30 nm). Even a thin 5 nm of Al_2_O_3_ barrier already reduces the maximum amplification to values below 400, similar to the experimental E.F. On the other hand, unfortunately, the pores (the air) occupy only a fraction of the membrane, so that the dielectric layer is an in-plane combination of alumina and air, which also modifies the interference response (Figure 6b). The estimated fraction of pores in the *h* = 100 nm sample obtained from the SEM images is around 20%. In Figure 6c both issues are combined and E.F. has been calculated for 20% pore fraction and different values of the alumina bottom barrier (0, 2 and 5 nm). The horizontal green line indicates the experimental E.F. obtained for the *h* = 100 nm membrane with good coincidence with 20% pore fraction and 0 nm alumina barrier. According to calculations the actual depth of the pores is around 90 nm rather than the 100 nm estimated from the SEM images and the treatment to reduce the bottom barrier is effective. 

**Figure 7:**
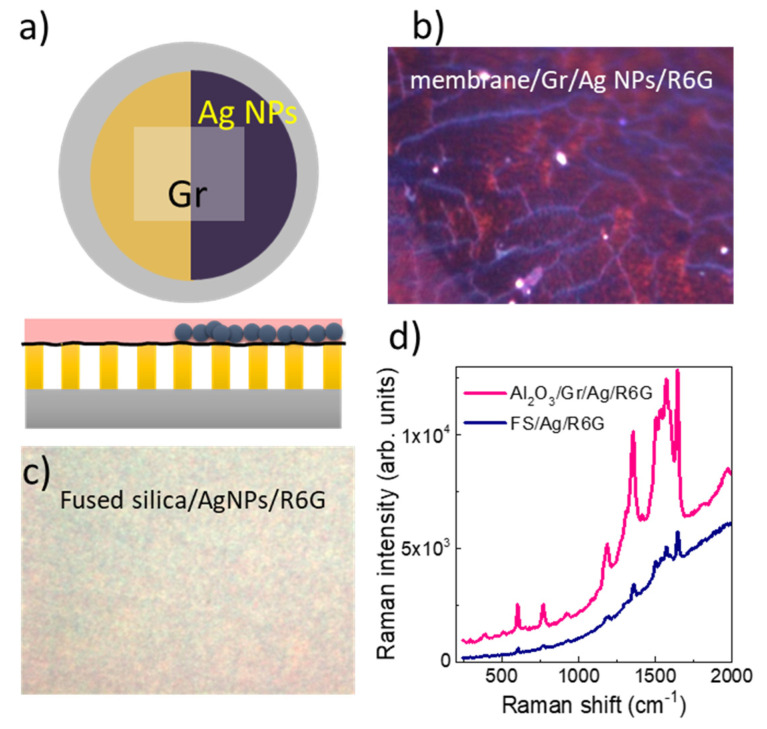
(**a**) Schema of the membrane based amplification platform: supported membrane/graphene/Ag NPs/R6G, top view and cross section, (**b**) optical image of the Ag NPs region and (**c**) of the fused silica/graphene/Ag NPs/R6G reference sample (images of 36 µm × 27 µm regions). (**d**) Typical R6G Raman spectra from both samples.

Finally, 4 nm silver nanoparticles (NPs) were deposited [see Refs. 4 and 34 for the NPs characterization] onto the graphene layer on top of the membrane to check the possibility to further enhance the Raman signal of graphene and of an analyte. A schema of the resulting membrane based system is plotted in Figure 7a). In this case we used Rhodamine 6G (R6G) to test the amplification capability. Ag NPs were simultaneously deposited on the membrane platform and on the reference sample (fused silica/graphene) previously used and R6G was then deposited by spin coating. Ultra-small Ag NPs were used to preserve the transparency required for the interference process and because we recently demonstrated that, for the same plasmon absorption, the SERS effect is more efficient for these 4 nm NPs than for larger ones due to higher hot-spot density [34]. 

Optical images of the membrane and the fused silica surfaces are shown in Figure 7b,c. The R6G fluorescence is almost quenched allowing to easily detect the R6G Raman modes. In Figure 7d, a representative Raman spectrum obtained for the membrane/graphene/Ag NPs/R6G (pink curve) is compared to that for the fused silica/graphene/Ag Nps/R6G sample (dark blue curve). A 10^−3^ M solution of R6G was deposited by spin coating on both substrate which correspond finally to the equivalent concentration of one R6G monolayer on the substrates [4]. The spectra present the characteristic Raman peaks of R6G. In both cases the same SERS and CM amplifications are occurring so that the intensity increase in the membrane spectra is due to the interference process demonstrating the cooperative amplification of SERS and interference effects. 

## 4. Conclusions

Supported alumina membranes where specifically designed and fabricated to be used as amplification platforms to enhance the Raman signal of analytes by interference processes of the incoming and scattered light beams. The metallic aluminum support is used as the reflecting medium and the dielectric layer is the combination of the air of the pores and the alumina of the walls. Pore diameters in the 10–20 nm range are adequate to transfer a CVD single-layer graphene to serve as the substrate to deposit the analyte to be detected. Graphene mimics the membrane surface but presents a flat surface with small height fluctuations ~3 nm, which is found to be adequate for interference efficiency. The theoretical optimum pore depth depends on the pore fraction of the membrane, it is around 60 nm for the first interference order and around 200 nm for the second order for 20% pore fraction. Platforms based on membranes with pores height around 60, 100 and 200 nm were prepared, however, the control of the membrane quality in the 60 nm range is not enough. The elimination of the alumina barrier layer (at the bottom of the pores) is crucial according to calculations and the employed process for its elimination is found to be totally efficient. The Raman signals of Rhodamine 6G, spin-coated on graphene and graphene itself were used to test the platforms. E.F. up to 400 is obtained for membranes with ~100 nm pore depth, ~18 nm pore diameter and the complete elimination of the Al_2_O_3_ bottom barrier layer. The most limiting parameter is the pore fraction in the membrane, which reaches around 20% for 18 nm pore diameter. Further pore widening, which is favorable to increase E.F. in principle, produces larger in-plane disorder and surface roughness (height distribution). 

We demonstrated the possibility to further enhance the Raman signal of R6G by depositing ultra-small (4 nm diameter) silver nanoparticles on the graphene layer prior to spin-coating the analyte. Combined SERS and IERS processes is observed. 

This new approach to amplify the Raman signal of analytes by means of interference is cheap and robust with useful enhancement factors (~400). It allows the combination of plasmonic and interference amplifications, however, these platforms are also appropriate to amplify Raman signals in the cases where the use of nanoparticles is to be avoided.
